# Efficacy, Safety, and Tolerability of Gepotidacin (GSK2140944) in the Treatment of Patients with Suspected or Confirmed Gram-Positive Acute Bacterial Skin and Skin Structure Infections

**DOI:** 10.1128/AAC.02095-16

**Published:** 2017-05-24

**Authors:** William O'Riordan, Courtney Tiffany, Nicole Scangarella-Oman, Caroline Perry, Mohammad Hossain, Teri Ashton, Etienne Dumont

**Affiliations:** a eStudySite, San Diego, California, USA; b GlaxoSmithKline, Upper Providence, Pennsylvania, USA; c GlaxoSmithKline, King of Prussia, Pennsylvania, USA

**Keywords:** ABSSSI, phase 2 study, antibacterial agent, antimicrobial safety, efficacy, gepotidacin, skin infections

## Abstract

Gepotidacin is a novel, first-in-class, triazaacenaphthylene antibacterial agent which has *in vitro* activity against causative pathogens of acute bacterial skin and skin structure infections (ABSSSIs). This phase 2, randomized, 2-part, multicenter, dose-ranging, response-adaptive study with optional intravenous-oral switch evaluated the efficacy and safety of gepotidacin for the treatment of Gram-positive ABSSSIs in 122 adult patients in the United States. The study had a double-blind phase (part 1; intravenous [750 mg or 1,000 mg every 12 h {q12h}]) and an open-label phase (part 2; intravenous [750 mg q12h, 1,000 mg q12h, or 1,000 q8h]). The primary endpoint was a composite of efficacy and safety which consisted of the early cure rate and the withdrawal rate due to drug-related adverse events and utilized a clinical utility index for dose selection. At the early efficacy visit (48 to 72 h after the first dose), the 750-mg q12h and 1,000-mg q8h groups met prespecified success criteria for clinical utility in terms of efficacy and safety; however, the 1,000-mg q12h group did not meet these criteria due to observed lower efficacy rates. The most frequently reported adverse events were nausea (20%) and diarrhea (13%). These encouraging phase 2 results demonstrate the potential for gepotidacin to meet the medical need for novel antibacterial agents to treat ABSSSIs due to drug-resistant pathogens through a unique mechanism of action. (This study has been registered at ClinicalTrials.gov under registration no. NCT02045797.)

## INTRODUCTION

An increasing number of patients are seeking treatment for acute bacterial skin and skin structure infections (ABSSSIs) (1–4). Given the increasing incidence of resistance to established antibiotic therapies, particularly in staphylococci, a need exists for an antibiotic with a novel mechanism of action that is safe and effective against drug-resistant ABSSSI pathogens and is available in both intravenous and oral formulations (5–10).

Gepotidacin is a novel, first-in-class, triazaacenaphthylene antibacterial agent that inhibits type IIA topoisomerases and has demonstrated *in vitro* activity against a broad spectrum of bacterial species, including methicillin-resistant Staphylococcus aureus (MRSA) and other primary causative pathogens of ABSSSIs (11, 12). The structure and mechanism of action of gepotidacin are unique, which allows gepotidacin to retain activity against target pathogens that are resistant to other antimicrobial agents, including fluoroquinolones (11). Gepotidacin inhibits bacterial DNA replication through a unique interaction on the bacterial subunits of DNA gyrase (GyrA) and topoisomerase IV (ParC). The stabilized equilibrium state of gepotidacin is associated with uncleaved and single-stranded cleaved DNA complexes (11). Gepotidacin has been evaluated in healthy human subjects by use of both intravenous and oral formulations (13).

The primary objective of this study was to identify the optimal dose of gepotidacin by evaluating the clinical efficacy and safety of gepotidacin in adult patients with suspected or confirmed Gram-positive ABSSSIs. The secondary objectives included determining the dose-related clinical efficacy, safety, tolerability, and pharmacokinetics of gepotidacin.

## RESULTS

### Patient and lesion baseline characteristics.

The majority of patients were white, middle-aged men (mean age, 44.7 years), with a mean body mass index of 27.33 kg/m^2^ (Table 1). Of the 126 randomized patients, 122 patients received study treatment and were included in the modified intent-to-treat (mITT) and safety populations, while 4 patients discontinued the study before receiving study treatment (Table 2). The mean exposure to intravenous gepotidacin was 3.4 days overall, and the mean exposure to oral gepotidacin was 7.5 days overall.

**TABLE 1:** Demographics and baseline characteristics (mITT population)^*a*^

Characteristic	Value or description			
	750-mg q12h group (*n* = 58)	1,000-mg q12h group (*n* = 39)	1,000-mg q8h group (*n* = 25)	Total (*n* = 122)
Age (yr)				
Mean (SD)	44.3 (11.35)	44.6 (10.76)	45.8 (12.64)	44.7 (11.36)
Minimum, maximum	19, 68	21, 71	22, 78	19, 78
No. (%) of patients in age group				
18 to 64 yr	56 (97)	38 (97)	24 (96)	118 (97)
65 to 74 yr	2 (3)	1 (3)	0	3 (2)
≥75 yr	0	0	1 (4)	1 (<1)
No. (%) of male patients	40 (69)	27 (69)	20 (80)	87 (71)
No. (%) of patients who were not Hispanic or Latino	35 (60)	16 (41)	15 (60)	66 (54)
Mean (SD) body mass index (kg/m^2^)	27.66 (5.186)	27.18 (4.140)	26.80 (3.910)	27.33 (4.605)
No. (%) of infections of ABSSSI type				
Wound infection	27 (47)	18 (46)	9 (36)	54 (44)
Major cutaneous abscess	17 (29)	16 (41)	6 (24)	39 (32)
Cellulitis	14 (24)	5 (13)	10 (40)	29 (24)
Baseline lesion pathogens				
No. of pathogens recovered	55	38	18	111
No. (%) of individual pathogens^*b*^				
Staphylococcus aureus	39 (71)	28 (74)	11 (61)	78 (70)
MRSA	29 (53)	20 (53)	5 (28)	54 (49)
MSSA	10 (18)	8 (21)	6 (33)	24 (22)
Other Gram-positive aerobic pathogens^*c*^	4 (7)	4 (11)	3 (17)	11 (10)

a ABSSSI, acute bacterial skin and skin structure infection; mITT, modified intent to treat; MRSA, methicillin-resistant S. aureus; MSSA, methicillin-susceptible S. aureus; q8h, every 8 h; q12h, every 12 h.

b Pathogen recovery rate (the percentage refers to the number of pathogens divided by the total number of pathogens recovered for the group).

c Other Gram-positive aerobic pathogens include the following: beta-hemolytic streptococcus groups A, F, and G, Staphylococcus epidermidis, Staphylococcus lugdunensis, and viridans group streptococci.

**TABLE 2:** Summary of study treatment statuses (mITT population)^*a*^

Completion status or reason for discontinuation	No. (%) of patients in treatment group			
	750 mg q12h (*n* = 58)	1,000 mg q12h (*n* = 39)	1,000 mg q8h (*n* = 25)	Total (*n* = 122)
Completed	51 (88)	32 (82)	24 (96)	107 (88)
Discontinued^*b*^	7 (12)	7 (18)	1 (4)	15 (12)
Primary reason for discontinuation				
Adverse event	3 (5)	1 (3)	0	4 (3)
Lack of efficacy	1 (2)	0	0	1 (<1)
Lost to follow-up	2 (3)	3 (8)	1 (4)	6 (5)
Physician decision	0	1 (3)	0	1 (<1)
Withdrawal by patient	1 (2)	2 (5)	0	3 (2)

a mITT, modified intent to treat; q8h, every 8 h; q12h, every 12 h.

b Only 1 patient withdrew due to a drug-related event.

**FIG 1:**
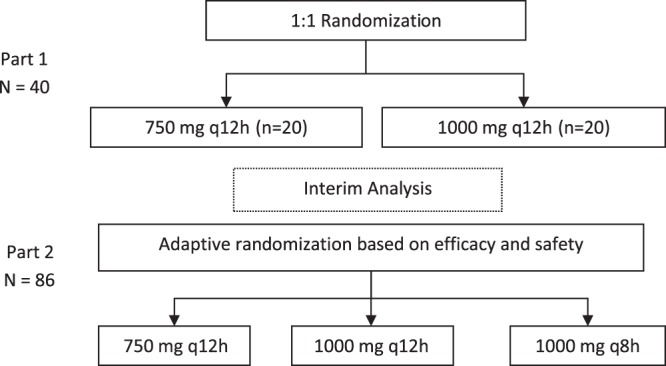
Study design. In both part 1 and part 2, at the discretion of the investigator, patients who completed the minimum intravenous dosing duration could be switched to a corresponding unblinded oral dosing regimen (1,500 mg or 2,000 mg twice a day, or 2,000 mg three times a day for part 2) in an outpatient setting to complete 10 days of total treatment. Part 2 was expected to enroll approximately 80 patients by use of an adaptive randomization strategy. A total of 86 patients were enrolled in part 2. Each of the preidentified doses for part 2 were used based on the outcomes of the analyses from part 1. The maximum dose was not to exceed a total daily dose of 4.5 g/day given intravenously or 6.0 g/day given orally.

**FIG 2:**
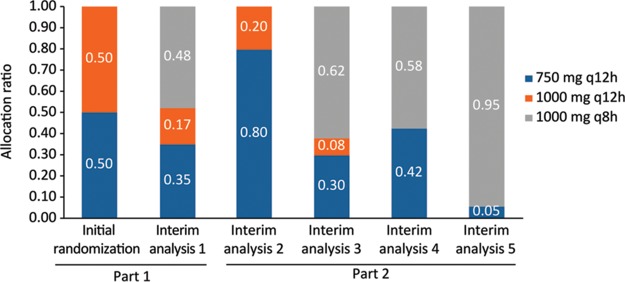
Adaptive randomization allocation ratios over time following each interim analysis (all-randomized population). The initial randomization bar represents part 1 of the study, where there was 1:1 randomization allocation to the 750-mg q12h and 1,000-mg q12h treatment groups. Interim analysis 5 represents the randomization allocation should the study have continued further.

After the 40-patient run-in period (part 1) with 1:1 randomization to 2 lower doses, patients were preferentially randomized to optimal doses (Fig. 1). Figure 2 shows how patients were allocated to groups over time, based on their overall clinical utility value at any given point, and Table 3 provides the supporting efficacy and safety data on which the utilities were based. The adaptive randomization allocated approximately half of the patients throughout the study to the treatment group receiving 750 mg every 12 h (q12h). If the study continued, the fifth adaptation would have allocated the majority of patients to receive 1,000 mg q8h, a small proportion of patients to receive 750 mg q12h, and no patients to receive 1,000 mg q12h.

**TABLE 3:** Primary composite endpoint (mITT population) at each analysis

Parameter	Value for treatment group														
	Interim analysis 1			Interim analysis 2			Interim analysis 3			Interim analysis 4			Final analysis		
	0.75g q12h	1 g q12h	1 g q8h	0.75g q12h	1 g q12h	1 g q8h	0.75g q12h	1 g q12h	1 g q8h	0.75g q12h	1 g q12h	1 g q8h	0.75g q12h	1 g q12h	1 g q8h
No. of patients	18	18	0	23	23	6	36	32	13	46	37	13	58	39	25
No. of patients who completed treatment^*a*^	18	18	0	23	23	6	36	31	13	46	37	13	58	39	25
No. of patients cured^*b*^	17	14	0	21	17	3	31	23	11	41	27	11	48	28	23
Modeled cure rate	0.83	0.78	0.52	0.83	0.74	0.5	0.81	0.74	0.85	0.84	0.73	0.85	0.80	0.72	0.92
New allocation ratio	0.35	0.17	0.48	0.80	0.20	0	0.30	0.08	0.62	0.42	0	0.58	0.05	0	0.95

a Includes all patients who completed the primary efficacy evaluation at day 3 or prematurely discontinued treatment.

b Includes all patients who achieved clinical success at the early efficacy visit, based on the sponsor-determined clinical outcome.

Overall, most of the patients had a wound infection (44%), followed by patients with a major cutaneous abscess (32%) or cellulitis (24%). The largest proportion of major cutaneous abscesses occurred in the 1,000-mg q12h treatment group (41%), and the largest proportion of cellulitis (40%) occurred in the 1,000-mg q8h treatment group. Based on digital image measurements, the overall mean (standard deviation [SD]) lesion size was 345.5 cm^2^ (322.59 cm^2^). The 750-mg q12h treatment group had the largest mean (SD) lesion size, 397.2 cm^2^ (389.37 cm^2^), followed by lesion sizes of 377.5 cm^2^ (299.47 cm^2^) and 248.2 cm^2^ (181.17 cm^2^) for the 1,000-mg q8h and 1,000-mg q12h treatment groups, respectively. (Additional details regarding lesion size are provided in the supplemental material.) Purulence and lymphadenopathy were present in the majority of patients (74% and 84%, respectively).

Gepotidacin was active *in vitro* against S. aureus isolates recovered from baseline lesion samples, with median MIC and MIC_90_ values of 0.25 μg/ml and 0.5 μg/ml, respectively. With the exception of 2 MRSA isolates, which had gepotidacin MIC values of 8 μg/ml and >32 μg/ml and were recovered from baseline lesion samples from 2 patients, all S. aureus isolates were inhibited at gepotidacin concentrations of ≤2 μg/ml. Molecular characterization of the 2 MRSA isolates with elevated gepotidacin MICs indicated that both strains harbored the following mutations: ParC S80Y, ParE D422E, GyrA S84L, and GyrA D83N. The MRSA isolate with the gepotidacin MIC of >32 μg/ml had an additional mutation in ParC (V67A). Two patients had a positive blood culture at baseline; both cultures were positive for MRSA, and both patients were in the 750-mg q12h treatment group.

### Efficacy. (i) Composite cure and withdrawal rates.

At the early efficacy visit, the 750-mg q12h and 1,000-mg q8h treatment groups had mean final utilities of 1.8225 and 2.5152, respectively, with a posterior probability of >85% for a clinical utility index (CUI) of >1.1 (Table 4). These 2 treatment groups met the primary endpoint criteria, and the study was considered a success. In addition, the 1,000-mg q8h treatment group had a posterior probability for a CUI of >1.8 that was also >85%, indicating that this treatment group was highly successful and represented the best dose from a statistical perspective. Patients in the 1,000-mg q8h treatment group were more likely than those in the other treatment groups to have a cure rate above 80% (Table 4). The 1,000-mg q12h treatment group had a mean final utility of 1.3124; however, the posterior probability for a CUI of >1.1 was <85%, which did not meet the protocol-specified criteria for having clinically significant utility.

**TABLE 4:** Primary composite endpoint (mITT population)^*a*^

Parameter	Value for treatment group		
	750 mg q12h	1,000 mg q12h	1,000 mg q8h
No. of treated patients	58	39	25
No. of patients who completed treatment^*b*^	58	39	25
No. of patients cured^*c*^	48	28	23
Mean (SD) raw cure rate	0.8276 (0.05)	0.7179 (0.073)	0.92 (0.0554)
Mean (SD) modeled cure rate	0.8043 (0.043)	0.718 (0.0714)	0.9195 (0.0531)
No. of drug-related withdrawals	1	0	0
Mean (SD) raw withdrawal rate	0.0172 (0.0172)	0 (0)	0 (0)
Mean (SD) modeled withdrawal rate	0.0131 (0.0093)	0.0093 (0.0083)	0.0056 (0.0082)
Posterior mean utility (SD) for cure rate	1.8237 (0.2621)	1.3129 (0.4016)	2.517 (0.3191)
Posterior mean utility (SD) for withdrawal rate	0.9993 (0.0054)	0.9997 (0.004)	0.9993 (0.0159)
Posterior mean (SD) of final utility	1.8225 (0.2621)	1.3124 (0.4015)	2.5152 (0.3214)
Pr(*P_d_* > CUI of 1.1)^*d*^	0.991964	0.67066	0.998268
Pr(*P_d_* > CUI of 1.8)^*e*^	0.562476	0.122688	0.965128
Pr(given dose is the one with maximum utility)	0.054384	0.008128	0.937488
New allocation ratio	0.0548	0	0.9452

a CUI, clinical utility index; mITT, modified intent to treat; *P_d_*, effective cure rate for dose *d*; Pr, probability; q8h, every 8 h; q12h, every 12 h. Note that patients were analyzed according to the actual treatment received.

b Includes all patients who completed the primary efficacy evaluation at day 3 or prematurely discontinued treatment.

c Includes all patients who achieved clinical success at the early efficacy visit, based on the sponsor-determined clinical outcome.

d The study was considered to have met its primary endpoint if a dose had a CUI of >1.1, a threshold corresponding to approximately 75% cure and 2.5% withdrawal rates, with a high (>85%) Bayesian posterior probability.

e A CUI of >1.8 was also used for exploratory superiority testing of the doses.

### (ii) Sponsor-determined clinical response.

**FIG 3:**
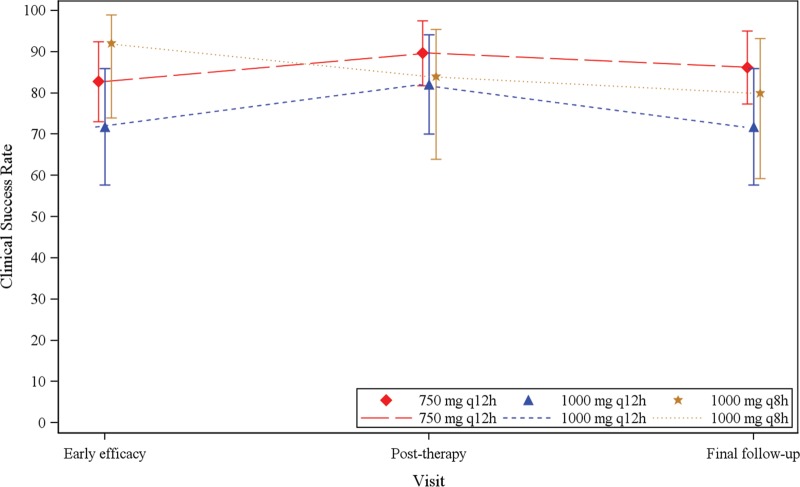
Clinical response (with 95% confidence interval) by dose group (mITT population). Mean success rates for the early efficacy visit were as follows: 750-mg q12h group, 83%; 1,000-mg q12h group, 72%; and 1,000-mg q8h group, 92%. Mean success rates for the posttherapy visit were as follows: 750-mg q12h group, 90%; 1,000-mg q12h group, 82%; and 1,000-mg q8h group, 84%. Mean success rates for the final follow-up visit were as follows: 750-mg q12h group, 86%; 1,000-mg q12h group, 72%; and 1,000-mg q8h group, 80%.

For both the 750-mg q12h and 1,000-mg q8h treatment groups, 80% or more of patients were considered to represent sponsor-determined clinical successes at all assessment visits (Table 5). The 1,000-mg q12h treatment group had the smallest number of clinical successes, with 72% at the early efficacy visit, 82% at the posttherapy visit, and 72% at the final follow-up visit. The clinical responses (with 95% confidence intervals) by dose group (mITT population) are shown in Fig. 3. Similar results were observed when sponsor-determined clinical successes were analyzed according to the pathogen isolated at baseline (additional details are provided in the supplemental material), ABSSSI type, and previous antibacterial drug therapy. Both patients with a positive baseline blood culture showed sponsor-determined clinical success.

**TABLE 5:** Summary of sponsor-determined clinical responses and outcomes (mITT population)^*a*^

Clinical visit and outcome	Value for treatment group			
	750 mg q12h (*n* = 58)	1,000 mg q12h (*n* = 39)	1,000 mg q8h (*n* = 25)	Total (*n* = 122)
Early efficacy visit				
No. (%) of successes	48 (83)	28 (72)	23 (92)	99 (81)
95% CI	73.0, 92.5	57.7, 85.9	74.0, 99.0	74.2, 88.1
No. (%) of clinical successes	48 (83)	28 (72)	23 (92)	99 (81)
No. (%) of failures	10 (17)	11 (28)	2 (8)	23 (19)
95% CI	7.5, 27.0	14.1, 42.3	1.0, 26.0	11.9, 25.8
No. (%) of clinical failures	7 (12)	7 (18)	2 (8)	16 (13)
No. (%) of “unable to determine” results	3 (5)	4 (10)	0	7 (6)
Posttherapy visit				
No. (%) of successes	52 (90)	32 (82)	21 (84)	105 (86)
95% CI	81.8, 97.5	70.0, 94.1	63.9, 95.5	79.9, 92.2
No. (%) of clinical successes	52 (90)	32 (82)	21 (84)	105 (86)
No. (%) of failures	6 (10)	7 (18)	4 (16)	17 (14)
95% CI	2.5, 18.2	5.9, 30.0	4.5, 36.1	7.8, 20.1
No. (%) of clinical failures	1 (2)	1 (3)	1 (4)	3 (2)
No. (%) of “unable to determine” results	5 (9)	6 (15)	3 (12)	14 (11)
Final follow-up visit	50 (86)	28 (72)	20 (80)	98 (80)
No. (%) of successes	77.3, 95.1	57.7, 85.9	59.3, 93.2	73.3, 87.4
95% CI	50 (86)	28 (72)	20 (80)	98 (80)
No. (%) of clinical successes				
No. (%) of failures	8 (14)	11 (28)	5 (20)	24 (20)
95% CI	4.9, 22.7	14.1, 42.3	6.8, 40.7	12.6, 26.7
No. (%) of clinical failures	1 (2)	1 (3)	1 (4)	3 (2)
No. (%) of clinical recurrences	2 (3)	1 (3)	1 (4)	4 (3)
No. (%) of “unable to determine” results	5 (9)	9 (23)	3 (12)	17 (14)

a CI, confidence interval; mITT, modified intent to treat; q8h, every 8 h; q12h, every 12 h.

### Safety and tolerability.

The incidences of patients who experienced adverse events (AEs) were similar between the 750-mg q12h and 1,000-mg q8h treatment groups (71% and 72%, respectively) and lowest for the 1,000-mg q12h treatment group (64%) (Table 6). Adverse events considered related to study treatment occurred in 33%, 44%, and 47% of patients in the 1,000-mg q12h, 1,000-mg q8h, and 750-mg q12h treatment groups, respectively. Overall, 4 patients (3%) experienced an AE that led to study withdrawal (1 patient) or permanent discontinuation of study treatment (3 patients). Two of these AEs were serious AEs (SAEs) that were not considered related to study treatment and consisted of 1 fatal SAE of septic shock that led to study withdrawal (1,000-mg q12h treatment group; this patient represented a clinical failure) and 1 nonfatal SAE of cellulitis that led to discontinuation of study treatment (750-mg q12h treatment group; this patient represented a clinical success at the early efficacy and posttherapy visits but a clinical failure at the follow-up visit). The other 2 AEs that led to discontinuation of study treatment were migraine (severe in intensity; this patient represented a clinical success) and a positive blood culture for Staphylococcus species (this patient represented a clinical failure); both of these events occurred in patients who received gepotidacin at 750 mg q12h. Only the AE of migraine was considered related to study medication, even though the patient had a preexisting condition of migraine; therefore, this was the only AE that contributed to the primary composite endpoint from a safety perspective.

**TABLE 6:** Summary of AEs reported by ≥5% of patients in any treatment group (safety population)^*a*^

AE (preferred term)	No. (%) of patients with AE			
	750-mg q12h group (*n* = 58)	1,000-mg q12h group (*n* = 39)	1,000-mg q8h group (*n* = 25)	Total (*n* = 122)
Any event	41 (71)	25 (64)	18 (72)	84 (69)
Nausea	12 (21)	9 (23)	4 (16)	25 (20)
Diarrhea	5 (9)	6 (15)	5 (20)	16 (13)
ALT increased	4 (7)	3 (8)	2 (8)	9 (7)
Vomiting	3 (5)	2 (5)	3 (12)	8 (7)
AST increased	3 (5)	3 (8)	1 (4)	7 (6)
Headache	6 (10)	1 (3)	0	7 (6)
Flatulence	3 (5)	1 (3)	2 (8)	6 (5)
Gamma-glutamyltransferase increased	3 (5)	3 (8)	0	6 (5)
Infusion site extravasation	3 (5)	1 (3)	0	4 (3)
Pruritus, generalized	1 (2)	3 (8)	0	4 (3)
Gastroesophageal reflux disease	1 (2)	0	2 (8)	3 (2)

a ALT, alanine aminotransferase; AST, aspartate aminotransferase; q8h, every 8 h; q12h, every 12 h.

The most frequently reported AEs were nausea (20%) and diarrhea (13%). In the majority of cases, the onset of nausea was associated with intravenous treatment (72% of these patients had nausea on days 1 and 2). The incidences of these AEs were similar across treatment groups, with the exception of the 1,000-mg q8h treatment group, which was associated with increased incidences of diarrhea and vomiting (Table 6). The majority (51%) of all AEs were mild in intensity; 34% of treatment-related AEs were mild in severity. One patient in the 750-mg q12h treatment group experienced a severe treatment-related AE of migraine that led to discontinuation of study treatment; this was the only AE in the study that was considered to be severe in intensity.

### (i) Adverse events of special interest.

The incidence of rash was 3% (4 patients); these events were considered to be mild to moderate in intensity. The incidence of infusion site reaction events was 2% (2 patients); these AEs were considered to be mild in intensity. No patient experienced a cardiovascular AE during the study, and there were no dose-related trends observed for changes from baseline for electrocardiogram (ECG) parameters across treatment groups. None of the patients had a change from baseline QT duration corrected for heart rate by Fridericia's formula (QTcF) that was greater than 60 ms during the study. The overall incidence of gastrointestinal events, which were mild to moderate in intensity, was 39% (47 patients), with the highest incidence (56% [14 patients]) occurring in the 1,000-mg q8h treatment group, compared to 33% (19 patients) for the 750-mg q12h treatment group and 36% (14 patients) for the 1,000-mg q12h treatment group. The onset of gastrointestinal events was similar across treatment groups, with approximately 30% to 40% of patients having an onset within the first treatment day.

### (ii) Clinical laboratory evaluations.

There were no dose-related trends observed for clinical chemistry, hematology, or urinalysis laboratory evaluations across treatment groups. A total of 4 patients had elevated alanine aminotransferase (ALT) or aspartate aminotransferase (AST) values that were more than 3 times the upper limit of normal. Two of the 4 patients had a history of chronic hepatitis C, and the other 2 patients had a history of liver disease. With the exception of 2 patients, all patients had ALT or AST values that were less than 5 times the upper limit of normal, and all patients had values that were less than 10 times the upper limit of normal. No patient had liver function tests that met Hy's law (aminotransferase elevation accompanied by increased serum total bilirubin).

### Pharmacokinetics.

Mean plasma gepotidacin concentrations during intravenous administration were consistently higher across the entire profile as the dose increased from 750 mg q12h to 1,000 mg q12h and to 1,000 mg q8h (Table 7). Mean plasma gepotidacin concentrations during oral administration were also consistently higher across the entire profile as the dose increased from 1,500 mg twice a day (BID) to 2,000 mg BID. Mean concentrations generally remained above 0.5 μg/ml, the MIC_90_ for gepotidacin against S. aureus, over the dosing interval for both intravenous and oral administration. The mean extent of exposure over the dosing interval (area under the concentration-time curve over the dosing interval [AUC_0–τ_]) following oral dosing was comparable to that for the corresponding intravenous dose for each treatment group. All 3 dosing regimens achieved the predicted free, unbound-drug AUC (*f*AUC)/MIC stasis target of 13.4 for S. aureus isolates with gepotidacin MICs of ≤1 μg/ml and, as expected, were found to be efficacious against infections caused by these organisms.

**TABLE 7:** Summary of gepotidacin plasma pharmacokinetic parameters by treatment group, with outliers excluded (pharmacokinetic parameter population)^*a*^

Treatment group	Parameter	Value for dose type^*b*^	
		Intravenous	Oral
750 mg i.v. q12h or 1,500 mg oral BID	*n*	50	51
	*C*_max_ (ng/ml)	4,590 (42.4)	2,342 (84.7)
	*t*_max_ (h)^*c*^	1.95 (0.00, 3.05)	3.00 (0.95, 12.00)
	AUC_0–_*_t_* (ng·h/ml)	15,992 (36.7)	11,965 (101.7)
	AUC_0–τ_ (ng·h/ml)	16,159 (36.3)^*d*^	14,404 (78.2)^*e*^
1,000 mg i.v. q12h or 2,000 mg oral BID	*n*	33	33
	*C*_max_ (ng/ml)	6,059 (29.8)	4,329 (67.8)
	*t*_max_ (h)^*c*^	1.90 (0.90, 3.00)	2.92 (1.02, 12.00)
	AUC_0–_*_t_* (ng·h/ml)	20,904 (31.8)	19,308 (98.8)
	AUC_0–τ_ (ng·h/ml)	20,815 (31.7)	24,253 (65.4)^*f*^
1,000 mg i.v. q8h or 2,000 mg oral TID	*n*	24	24
	*C*_max_ (ng/ml)	8,848 (105.5)	3,858 (94.1)
	*t*_max_ (h)^*c*^	1.93 (0.00, 3.00)	2.98 (0.98, 8.00)
	AUC_0–_*_t_* (ng·h/ml)	20,851 (64.8)	15,923 (117.4)
	AUC_0–τ_ (ng·h/ml)	21,499 (66.9)^*g*^	19,300 (77.9)^*g*^

a AUC_0–_*_t_*, area under the plasma concentration-time curve from time zero to the last measurable concentration; AUC_0–τ_, area under the plasma concentration-time curve over the dosing interval (τ), where τ = 8 h for q8h or TID and τ = 12 h for q12h or BID; BID, twice a day; *C*_max_, maximum observed plasma concentration; i.v., intravenous; TID, 3 times a day; *t*_max_, time to reach *C*_max_; q8h, every 8 h; q12h, every 12 h. Note that blood samples for pharmacokinetic analysis were collected over a single dosing interval at predefined time points on day 1 or day 2 of intravenous dosing and upon administration of the first oral dose for analysis of gepotidacin concentrations. Outlier testing (Grubb's test) of i.v. pharmacokinetic parameters resulted in the exclusion from summary statistics of patient 000077 for 750 mg i.v. q12h or 1,500 mg oral BID and of patient 000101 for 1,000 mg i.v. q8h or 2,000 mg oral TID.

b Except for the *n* values, the data are geometric means (percent coefficients of variation) (unless stated otherwise).

c Data are medians (ranges).

d *n* = 49.

e *n* = 44.

f *n* = 28.

g *n* = 22.

## DISCUSSION

This phase 2, randomized, dose-ranging study of adult patients with ABSSSIs (ClinicalTrials registration no. NCT02045797) met its protocol-defined primary objective, which was a composite of efficacy (cure rate) and safety (withdrawal rate) that utilized a CUI for dose selection. The secondary endpoint of sponsor-determined clinical response also demonstrated clinical efficacy for all 3 dose regimens. These encouraging phase 2 results demonstrate the potential for gepotidacin to meet the medical need for novel antibacterial agents to treat ABSSSIs due to drug-resistant ABSSSI pathogens through a unique mechanism of action.

The statistical success of this study depended upon an optimal gepotidacin dose achieving a predefined utility of >1.1 with high probability at the early efficacy visit. Two treatment groups, the 750-mg q12h and 1,000-mg q8h groups, achieved the predefined criteria for successful treatment. The 1,000-mg q8h regimen was considered highly successful and was determined to be the optimal dosing regimen because it attained a predefined utility of >1.8 with high probability.

Although the 1,000-mg q12h treatment group had a mean final utility of 1.3124, it achieved this with a lower probability than was considered acceptable and therefore did not meet the protocol-specified criteria for having a clinically significant utility. The lower rate of clinical success for this mid-dose treatment group may have been the result of several potential contributing factors. First, with a small sample size of 39 patients and the imbalance between treatment groups, the success or failure for a single patient had a greater impact on the results and may have led to a lower posterior probability to meet the CUI. This treatment group had the highest incidence of the wound and major cutaneous abscess lesion types combined (87%) compared to the other treatment groups, as well as having the highest incidence of study discontinuations (18%), which led to more patients with “unable to determine” outcomes that were defined as clinical failures. Second, the higher clinical failure rate may have been attributed to ABSSSI lesions that were more difficult to treat (e.g., 1 patient had an SAE of septic shock after a single dose of gepotidacin, which led to study discontinuation, and 1 patient had a MRSA isolate with a gepotidacin MIC of >32 μg/ml, which was the highest observed gepotidacin MIC against S. aureus in the study). Third, the patients in this treatment group had the smallest mean lesion size at baseline and the lowest percentage of patients with cellulitis (13%). Therefore, a reduction in lesion size that was ≥20% (i.e., the definition for success) may have been more difficult to detect and to achieve due to the size proportion and possible measurement error. Overall, it is likely that all 3 dose levels tested in the study were near the top of the dose-response curve and that the lower efficacy rates observed at the mid-dose level were due to the potential reasons noted above, since no significant dosing errors were noted and the pharmacokinetic parameters were as predicted.

There were several preplanned interim analyses performed in this study, which adhered to a response-adaptive randomization design that allowed for regular safety monitoring and adjustment of the randomization allocation ratio. There were no untoward or unexpected safety concerns with the administration of gepotidacin in patients with ABSSSIs in this study, and no recommendations for changes in study conduct were made throughout the study by the internal safety review committee.

Previous studies indicated that gepotidacin has been associated with certain AEs that were predefined as events of special interest in the present study. These AEs included rash, infusion site reaction, cardiovascular (i.e., changes in corrected QT intervals), central nervous system, and gastrointestinal events, including Clostridium difficile-associated diarrhea (14, 15). In the current study, events of rash and infusion site reactions were uncommon. Infusion site reactions were managed by using study treatment solutions with a pH of 4 for BID dosing and a pH of 5 for dosing 3 times a day. Although there were no cardiovascular events reported in the present study, observations of QT duration corrected for heart rate by Bazett's formula or QTcF prolongation in this study were consistent with the known effects of gepotidacin (a 1,000-mg dose leads to a QTcF prolongation of approximately 12 ms) (14).

The occurrence of gastrointestinal events in this study, particularly nausea (20%) and diarrhea (13%), was not unexpected. However, these events were generally mild in intensity, and none of them led to study discontinuation. There were no dose-related trends in the frequency of central nervous system events, which were observed in only a few patients. One patient experienced an AE of migraine that was considered severe in intensity by the investigator; the patient had a preexisting condition of migraine. There was no occurrence of C. difficile-associated diarrhea in this study. Although some AEs reported in this study (e.g., nausea, diarrhea, and salivary hypersecretion) are associated with acetylcholinesterase inhibition, which is a potential effect of gepotidacin, none of these AEs led to study discontinuation for any patient (16).

Although limited blood samples were collected for pharmacokinetic analyses following intravenous and oral dosing, this study demonstrated that the pharmacokinetics in adult patients with ABSSSIs were generally comparable to those in healthy volunteers (13). Because the absolute oral bioavailability was previously determined to be approximately 50%, the oral doses in this study (1,500 mg and 2,000 mg) were 2-fold higher than the corresponding intravenous doses (750 mg and 1,000 mg, respectively), which provided comparable exposures following oral and intravenous dosing (13). An exposure-response relationship, even if present, would have been difficult to identify based on the following results or parameters of the study: achievement of the predicted *f*AUC/MIC stasis target with gepotidacin MICs of ≤1 μg/ml, efficacy of all 3 doses, pharmacokinetic variability in patients, a 2-fold difference in total daily doses for the 3 dose regimens, few failures, and general tolerability of all doses.

The clinical utility index was based on success rates at the early efficacy assessment and withdrawal rates due to AEs deemed related to study treatment. As only 1 patient met the safety endpoint, the clinical utility of the 3 doses evaluated in the study was essentially based on the efficacy endpoint. The 3 doses did not differentiate well based on efficacy, likely due to their being at the top of the dose-response curve. The 1,000-mg q12h group had the lowest response rate and was determined to have not met the protocol-specified criteria for having clinical utility; however, the lower success rate was potentially driven by factors not related to the dose of gepotidacin. Regardless, the response-adaptive randomization design, which utilized a composite endpoint, performed well and did allow for the preferential randomization of patients to treatment arms with the best utility throughout the conduct of the trial. The Bayesian decision rules based on the utility were used effectively for the evaluation of futility, allocation to treatment arms, and final dose selection. The composite endpoint allowed simultaneous evaluations of efficacy and safety parameters and offered the opportunity to differentiate doses objectively based on both efficacy and safety parameters. This study design likely contributed to the success of the study by allowing for adjustments based on emerging results. Future studies of antibacterial agents may benefit from the use of the response-adaptive randomization approach.

Interpretations of the results of this study may be limited by several factors. First, portions of this study were open label. In part 1, intravenous treatment was double blinded; however, when a patient switched to oral therapy (after day 3), the treatment was open label. All of part 2 of the study was open label. Second, there was no comparator agent in this study, and no conclusions can be drawn regarding comparisons of the clinical efficacy or safety of gepotidacin and that of other antimicrobial agents. Finally, no conclusions can be drawn regarding the efficacy and safety of gepotidacin in patients with less severe ABSSSIs (i.e., not requiring hospitalization) and in other countries, as this study was conducted exclusively at study centers in the United States (additional discussion points are addressed in the supplemental material).

### Conclusions.

In summary, this study demonstrated that both the intravenous and oral routes of administration of gepotidacin appear to be safe and effective for the treatment of patients with suspected or confirmed Gram-positive ABSSSIs. Statistically, the study met its protocol-defined primary objective and endpoint for the 750-mg q12h (oral dose, 1,500 mg) and 1,000-mg q8h (oral dose, 2,000 mg) treatment groups but not for the 1,000-mg q12h gepotidacin treatment group. Several confounding factors may have contributed to this result, including an imbalance in lesion types and a higher discontinuation rate for the last treatment group. The administration of gepotidacin was generally well tolerated in patients with ABSSSIs in all 3 treatment groups, with no unexpected findings. These phase 2 data suggest that gepotidacin, a new antimicrobial agent with a novel mechanism of action, has the potential to address a critical need for novel antibacterial agents for the treatment of ABSSSIs caused by targeted drug-resistant pathogens.

## MATERIALS AND METHODS

### Patients.

Adult (≥18 years of age) male and female (nonpregnant) patients were eligible for enrollment into the study if they had a diagnosis of ABSSSI with a minimum surface area of 75 cm^2^ that was suspected or documented to be caused by a Gram-positive pathogen and required intravenous antibiotic treatment in an inpatient setting for at least 2 days. The original protocol inclusion criteria required patients to have a rapid diagnostic skin swab test positive for S. aureus, which limited the ABSSSI lesion types qualified for patient enrollment to wound infections and major cutaneous abscesses. In order to broaden the lesion types eligible for enrollment, amendment 3 to the protocol expanded the definition of ABSSSI to include cellulitis and removed the requirement for the rapid diagnostic skin swab test positive for S. aureus (additional details are provided in the supplemental material).

### Dose selection.

To predict the ability of potential gepotidacin doses to achieve the *f*AUC/MIC stasis target of 13.4, which was determined from a pharmacokinetic/pharmacodynamic neutropenic murine thigh model (17), a population pharmacokinetic model was developed using the concentration-time data for intravenous dosing. Pharmacokinetic variability in drug clearance was inflated to 40% to mimic that of an infected patient population. Monte Carlo simulations indicated that >90% of the simulated patients were expected to achieve the *f*AUC/MIC stasis target of 13.4 for S. aureus isolates with gepotidacin MICs of ≤1 μg/ml for the 750-mg and 1,000-mg intravenous dose regimens. The absolute oral bioavailability was previously determined to be approximately 50%; therefore, the oral doses (1,500 mg and 2,000 mg) were 2-fold higher than the corresponding intravenous doses (750 mg and 1,000 mg).

### Study design.

This phase 2, randomized, 2-part, multicenter, dose-ranging, response-adaptive study evaluated the clinical efficacy, safety, tolerability, pharmacokinetics, and pharmacodynamics of gepotidacin therapy for the treatment of suspected or confirmed Gram-positive ABSSSIs in adult patients in 13 medical centers in the United States. The study was divided into a double-blind intravenous dosing–open-label oral dosing phase (part 1) and an unblinded intravenous–open-label oral phase (part 2) (Fig. 1). The study employed a response-adaptive randomization design based on a utility function which incorporated a composite of safety and efficacy. Multiple interim analyses were performed with the aim of adjusting the allocation ratio to preferentially randomize patients to the dose with the best overall outcome in terms of efficacy and safety. The first interim analysis occurred at the end of part 1 to obtain an initial understanding of activity and to confirm the dose regimens for part 2, after approximately 40 patients had been randomly assigned, completed the early efficacy visit, and completed treatment. Thereafter, additional interim analyses occurred after approximately every 20 patients were enrolled in part 2 of the study. The potential outcomes of the part 2 interim analyses were to stop the study because of futility, continue randomization of patients per protocol, or increase the overall number of patients enrolled in the study (up to a maximum of 160 patients).

In part 1, patients were randomized 1:1 into 2 gepotidacin treatment groups (750 mg or 1,000 mg q12h), with the drug administered intravenously for a minimum of 48 to 72 h in an inpatient setting. Patients could be switched to corresponding open-label oral doses at the investigator's discretion after the minimum of 48 to 72 h of intravenous dosing.

In part 2, after an interim analysis, patients were adaptively randomized based on the cure and withdrawal rates observed in part 1 into 3 unblinded, open-label, gepotidacin treatment groups (750 mg q12h, 1,000 mg q12h, and 1,000 mg q8h), with the drug administered intravenously for a minimum of 48 h in an inpatient setting (Fig. 1). The duration of the study was approximately 3 to 4 weeks. An interactive voice response system monitored enrollment to ensure that no more than approximately 30% of patients who enrolled had major cutaneous abscesses.

### Ethical approval.

Written informed consent was obtained from all patients, and the study was conducted in accordance with good clinical practice as defined by the International Council for Harmonisation. The protocol, amendments, and patient-informed consent were approved by a local or academic institutional review board prior to initiation of the study.

### Study outcomes.

The primary endpoint was a composite of the cure rate, as measured by the clinical response and outcome at the early efficacy visit (48 to 72 h after the first dose of study treatment), and a safety component that consisted of all drug-related adverse events (AEs) that led to withdrawal before completion of therapy for all patients who received at least 1 dose of gepotidacin (additional details are provided in the supplemental material). Secondary efficacy endpoints included clinical response and outcome. Clinical response was determined programmatically based on the recorded clinical outcome and was used to determine clinical success, defined as a reduction in lesion size of at least 20%. Assessments of clinical efficacy were performed at the early efficacy, posttherapy (day 12 to 18), and final follow-up (day 21 to 28) visits.

An internal safety review committee oversaw the study and reviewed efficacy and safety on an ongoing basis. The safety assessments included the following: monitoring of AEs (including predefined AEs of special interest, namely, rash, infusion site reactions, cardiovascular events, and gastrointestinal effects), clinical laboratory tests, monitoring of vital signs, and electrocardiograms (ECGs). Safety evaluations were performed at all visits (daily while on intravenous therapy as an inpatient, at scheduled visits while on oral therapy as an outpatient, and after therapy). Blood samples for pharmacokinetic analysis were collected over a single dosing interval at predefined time points on day 1 or day 2 of intravenous dosing and upon administration of the first oral dose for analysis of gepotidacin concentrations.

### Microbiological assessments.

At baseline, a pretreatment bacteriology sample of the infected lesion was obtained by tissue biopsy, needle aspiration, or skin swab (if deemed most appropriate by the investigator) for culture and susceptibility testing. Blood cultures were also performed for all patients at baseline. A bacteriology lesion sample was obtained at any postbaseline visit at which culturable material was present, and positive blood cultures were repeated until a negative result was obtained. Bacteriology lesion samples and blood cultures were sent to a local laboratory for Gram staining, culture, and pathogen identification. All protocol-defined pathogens were sent to a central laboratory for confirmation of identification, and susceptibility testing was performed on all Gram-positive aerobic pathogens.

### 3D digital imaging assessments.

After a lesion was determined to be eligible for the study based on manual measurement, a baseline 3-dimensional (3D) digital image of the lesion was taken. The total surface area of the lesion was calculated based on the digital imaging. The assessment of clinical outcome was determined programmatically based on computer analysis of 3D digital images of the lesion. Digital images were processed and analyzed centrally to calculate the sponsor-determined clinical response.

### Statistical analyses.

A sample size of 120 patients for inclusion in the primary analysis was planned. The modified intent-to-treat (mITT) population consisted of all randomly assigned patients who received at least 1 dose of study medication. This population was the primary analysis population for efficacy and safety analyses and drove the decisions on adaptive randomization.

The primary endpoint was a composite of efficacy (clinical response rate at the early efficacy visit) and withdrawal (including all AEs considered to be drug related which led to withdrawal before completion of therapy) rates and was expressed as the clinical utility index (CUI). Specifically, the study was considered to have met its primary endpoint if a dose had a CUI of >1.1, a threshold corresponding to approximately 75% cure and 2.5% withdrawal rates, with a high (>85%) Bayesian posterior probability. A CUI of >1.8 was also used for exploratory superiority testing of the doses (additional details are provided in the supplemental material). The percent change from baseline of the total surface area of the lesion was summarized by use of descriptive statistics, by visit and dose group, for the mITT population.

Extensive simulations were performed to understand the trial operating characteristics. Assuming an 80% cure rate and a 5% withdrawal rate due to a gepotidacin-related AE for all doses studied, there was an approximately 71% chance that at least 1 dose would meet the clinical utility criteria for a total sample size of 120 patients. The chance improved to approximately 78% if the cure rate was 85% and improved further, to approximately 96%, if the withdrawal rate was 2%. Assuming a 45% cure rate and a 5% withdrawal rate due to a gepotidacin-related AE for all doses studied, there was a nearly 0% chance of 1 or more doses being erroneously declared efficacious.

Patient safety data (AEs and SAEs) were summarized and reported as numbers and percentages. The individual plasma-concentration versus actual time data for gepotidacin were used to derive the pharmacokinetic parameters by using noncompartmental pharmacokinetic methods.

## Supplementary Material

Supplemental material
