# Supplementary material for: Efficacy, Safety, and Tolerability of Gepotidacin (GSK2140944) in the Treatment of Patients with Suspected or Confirmed Gram-Positive Acute Bacterial Skin and Skin Structure Infections
Source: Antimicrob Agents Chemother. 2017 May 24;61(6):e02095-16. doi: 10.1128/AAC.02095-16 (PMC5444153; doi:10.1128/AAC.02095-16)
Supplement: Supplemental material [file supp_61_6_e02095-16__index.html]

Efficacy, Safety, and Tolerability of Gepotidacin (GSK2140944) in the Treatment of Patients with Suspected or Confirmed Gram-Positive Acute Bacterial Skin and Skin Structure Infections — Supplemental material 

# Efficacy, Safety, and Tolerability of Gepotidacin (GSK2140944) in the Treatment of Patients with Suspected or Confirmed Gram-Positive Acute Bacterial Skin and Skin Structure Infections

## Supplemental material

- Supplemental file 1 -

  Supplemental Material 1

  PDF, 290K
