# Supplementary material for: Efficacy, Safety, and Tolerability of Gepotidacin (GSK2140944) in the Treatment of Patients with Suspected or Confirmed Gram-Positive Acute Bacterial Skin and Skin Structure Infections
Source: Antimicrob Agents Chemother. 2017 May 24;61(6):e02095-16. doi: 10.1128/AAC.02095-16 (PMC5444153; doi:10.1128/AAC.02095-16)
Supplement: Supplemental material [file AAC.02095-16_zac006176239s1.pdf]

SUPPLEMENTAL MATERIALS

Title: **The Efficacy, Safety, and Tolerability of Gepotidacin (GSK2140944) in the Treatment of Patients with Suspected or Confirmed Gram-Positive Acute Bacterial Skin and Skin Structure Infections**

Authors: William O'Riordan, Courtney Tiffany, Nicole Scangarella-Oman, Caroline Perry, Mohammad Hossain, Teri Ashton, Etienne Dumont

METHODS

Patients

A wound infection (traumatic or postsurgical) was defined as an infection involving skin and subcutaneous tissue, characterized by purulent drainage from a wound with surrounding redness, edema, and/or induration of a minimum surface area of 75 cm^2 (eg, the shortest distance of redness, edema, and/or induration extending at least 5 cm from the peripheral margin of the wound). A major cutaneous abscess was defined as an infection characterized by a collection of pus within the dermis or deeper that was accompanied by redness, edema, and/or induration of a minimum surface area of 75 cm^2 (eg, the shortest distance of redness, edema, and/or induration extending at least 5 cm from the peripheral margin of the abscess). Cellulitis was defined as a diffuse skin infection characterized by a spreading area of redness, edema, and/or induration of a minimum surface area of 75 cm^2.

Patients also had at least 1 additional sign or symptom of skin infection (eg, fluctuation, heat or localized warmth, or pain/tenderness) and 1 systemic marker of infection (eg, lymphadenopathy, fever, white blood cell elevation, or C-reactive protein greater than the upper limit of normal),

unless the patient was older than 70 years of age or had known or suspected diabetes. Patients were excluded from the study if they had an immune-compromising illness, body mass index greater than or equal to 40.0 kg/m^2, a medical condition or required medication that may have been aggravated by inhibition of acetylcholinesterase, or a diagnosis of *Clostridium difficile* infection.

Patients were excluded from the study if they had electrocardiogram abnormalities (ie, PR interval <120 msec or >220 msec, QT duration corrected for heart rate by Bazett's or Fridericia's formula [QTcB or QTcF, respectively] >450 msec or QTcB or QTcF >480 msec for patients with bundle branch block, or QRS duration <70 msec or >120 msec). Other exclusion criteria included elevated liver function tests or liver disease, severe impairment of renal function, chronic neutropenia, and select skin conditions or infections. Patients who had received more than a single dose of antibiotic within the past 4 days were also excluded from participating in the study with the following exception: Patient received a single dose of a short-acting (half-life <12 hours) antibacterial before the first dose of study treatment; patient failed a previous ABSSSI regimen (ie, at least 48 hours of treatment) and had documented lack of microbiological or clinical response to such therapy; or patient recently completed a treatment course with an antibacterial drug for an infection other than ABSSSI and the drug did not have antibacterial activity against bacterial pathogens that cause ABSSSI.

Amendments to Protocol (Key Changes)

There were 3 amendments to the original protocol. The original protocol inclusion criteria required patients to have a rapid diagnostic skin swab test positive for *Staphylococcus aureus*,

which limited the ABSSSI types qualified for patient enrollment to wound infection and major cutaneous abscess. The original protocol also had a minimum 3-day requirement for intravenous (IV) dosing. These requirements were maintained through Amendments 1 and 2. In order to broaden the wound types eligible for enrollment, Amendment 3 removed the requirement for the rapid diagnostic skin swab test positive for *S. aureus* and expanded the definition of ABSSSI to include cellulitis and those suspected or confirmed to be caused by a Gram-positive pathogen. Amendment 3 also shortened the requirement for IV dosing duration to 2 days and decreased the sample size from 240 patients to 120 patients with a potential extension of enrollment for a maximum of 160 patients.

Statistical Analyses

Cure rate and withdrawal rate were analyzed with independent dose-response models and jointly assessed using a utility function. The dose response for cure rate was modeled with a normal dynamic linear model with the parameters evolution described by a Gaussian random walk. The dose-response for the withdrawal rate was modeled with a 2-parameter logistic model assuming a monotonic change. The utility function had 2 components, a cure rate component, and a withdrawal rate component, which were combined multiplicatively to yield the final utility. (Supplement Figure 1).

The cure rate component gives a score of 0 to cure rates below a clinically meaningful threshold of 50% then gives increasing utility for rates above the threshold. Some curvature is present at the low end of the curve to reflect the acceptable but less desirable rates below 75%. The withdrawal rate component effectively gives “full credit” to withdrawal rates less than 2.5%,

then decreasing "credit" above 2.5%, decreasing slowly at first, then rapidly to a "no credit" case for withdrawal rate of 10% or above. The final utility function was calibrated such that a minimally significant clinical utility index (CUI) of 1.1 or greater was considered clinically desirable.

At each interim and final analysis posterior probabilities were calculated, which quantified the probability of the outcome, in this case a utility greater than 1.1, given the information known at that time. The posterior distribution of all parameters in the dose-response models were jointly estimated by the Markov Chain Monte Carlo techniques based on the modified intent-to-treat population. The posterior probability that the utility was greater than the minimally significant CUI of 1.1 was calculated. At the final analysis, the study was considered a success if there was a greater than 85% Bayesian posterior probability that the maximum utility dose achieved a utility greater than 1.1 (1.8 was used as an exploratory CUI testing for further superiority).

RESULTS

Demographics and Patient Baseline Characteristics

The overall exposure to gepotidacin for the majority of patients (58%) was 10 days and was similar across treatment groups. The mean treatment compliance was 84% overall, 80% for IV dosing, and 87% for oral dosing.

Lesion Size

Lesion size (based on the mean percent change from baseline) continued to decrease with each subsequent assessment for all treatment groups. The 1000 mg every 8 hours (q8h) treatment

group had the largest mean percent decrease at the early efficacy visit (-54.3%) followed by the 750 mg every 12 hours (q12h) and 1000 mg q12h treatment groups (-46.5% and -45.3%, respectively). The mean percent decreases were similar across treatments at the post-therapy and final follow-up visits. The 1000 mg q8h treatment group also had the highest incidence of 20% or greater reduction in lesion size from baseline at the early efficacy visit (92%) compared with the 750 mg q12h treatment group (84%) and the 1000 mg q12h treatment group (72%).

Sponsor-Determined Clinical Responses by Pathogen

Supplement Table 1 summarizes the sponsor-determined clinical success rates by pathogen isolated at baseline. The treatment group patterns of clinical success by pathogen were similar to the overall pattern of clinical success shown in Supplement Table 1.

Investigator-Determined Clinical Responses

The 750 mg q12h treatment group had the highest percentage of clinical successes (90%-97%), followed by the 1000 mg q8h treatment group (84%-96%) and the 1000 mg q12h treatment group (72%-87%). For all treatment groups, the investigator-determined clinical successes were higher than sponsor-determined clinical successes at the early efficacy visit. For the 750 mg q12h and 1000 mg q8h treatment groups, the clinical successes were also higher for investigator-determined clinical success than sponsor-determined clinical successes at the final follow-up visit.

ADDITIONAL DISCUSSION POINTS

More patients in this study had a wound infection (44%) than major cutaneous abscess (32%) or cellulitis (24%). In contrast, registration ABSSSI studies for other recently approved antibacterial agents had patient populations with an infection type distribution that ranged from approximately 40% to 50% with cellulitis or erysipelas, 20% to 31% with major cutaneous abscess, and 20% to 30% with wound infection (1-3). In the current study, due to the pathogen-specific (*S. aureus*) focus of the initial study design, cellulitis was not allowed as an eligible ABSSSI wound type until the third amendment to the study and after removal of the rapid diagnostic test for *S. aureus*, which may have contributed to the overall lower percentage of cellulitis and a higher percentage of wound infections. For future study design considerations, an assessment of the operational and patient population impacts of a pathogen-specific study design and the use of a rapid diagnostic screening tool should be accounted for early in study design discussions.

REFERENCES

1. **Merck & Co, Inc.** 2015. Sivextro prescribing information. Merck & Co, Inc, Whitehouse Station, NJ.

2. **Durata Therapeutics.** 2014. Dalvance prescribing information. Durata Therapeutics, Chicago, Il.

3. **The Medicines Company.** 2014. Orbactiv prescribing information. The Medicines Company, Parsippany, NJ.

Supplement Table 1. Summary of sponsor-determined clinical success rate by pathogen (mITT population)[a]

Visit/Pathogen	Number of Successes/Number of Subjects (Clinical Success Rate [%]) (95% CI)			
	**750 mg q12h N=58**	**1000 mg q12h N=39**	**1000 mg q8h N=25**	**Total N=122**
**Early Efficacy Visit**				
Staphylococcus aureus	31/37 (84)<br>(71.9, 95.7)	21/28 (75)<br>(59.0, 91.0)	11/11 (100)<br>(71.5, 100.0)	63/76 (83)<br>(74.4, 91.4)
MRSA	22/28 (79)<br>(63.4, 93.8)	14/20 (70)<br>(49.9, 90.1)	5/5 (100)<br>(47.8, 100.0)	41/53 (77)<br>(66.1, 88.6)
MSSA	10/10 (100)<br>(69.2, 100.0)	7/8 (88)<br>(47.3, 99.7)	6/6 (100)<br>(54.1, 100.0)	23/24 (96)<br>(78.9, 99.9)
Other Gram-positive aerobic pathogens	3/4 (75)<br>(19.4, 99.4)	3/4 (75)<br>(19.4, 99.4)	3/3 (100)<br>(29.2, 100.0)	9/11 (82)<br>(48.2, 97.7)
All Gram-positive aerobic pathogens	33/40 (83)<br>(70.7, 94.3)	22/29 (76)<br>(60.3, 91.4)	13/13 (100)<br>(75.3, 100.0)	68/82 (83)<br>(74.8, 91.1)
Gram-negative aerobic pathogens	7/8 (88)<br>(47.3, 99.7)	3/6 (50)<br>(11.8, 88.2)	3/3 (100)<br>(29.2, 100.0)	13/17 (76)<br>(50.1, 93.2)
Anaerobic pathogens	0/0	0/0	1/1 (100)<br>(2.5, 100.0)	1/1 (100)<br>(2.5, 100.0)
All pathogens	36/44 (82)<br>(70.4, 93.2)	23/32 (72)<br>(56.3, 87.5)	15/15 (100)<br>(78.2, 100.0)	74/91 (81)<br>(73.3, 89.3)
No pathogens	12/14 (86)<br>(57.2, 98.2)	5/7 (71)<br>(29.0, 96.3)	8/10 (80)<br>(44.4, 97.5)	25/31 (81)<br>(66.7, 94.6)
**Post-Therapy Visit**				
Staphylococcus aureus	33/37 (89)<br>(74.6, 97.0)	25/28 (89)<br>(71.8, 97.7)	8/11 (73)<br>(39.0, 94.0)	66/76 (87)<br>(79.2, 94.4)
MRSA	24/28 (86)<br>(67.3, 96.0)	17/20 (85)<br>(62.1, 96.8)	4/5 (80)<br>(28.4, 99.5)	45/53 (85)<br>(75.3, 94.5)

Visit/Pathogen	Number of Successes/Number of Subjects (Clinical Success Rate [%]) (95% CI)			
	**750 mg q12h N=58**	**1000 mg q12h N=39**	**1000 mg q8h N=25**	**Total N=122**
MSSA	10/10 (100) (69.2, 100.0)	8/8 (100) (63.1, 100.0)	4/6 (67) (22.3, 95.7)	22/24 (92) (73.0, 99.0)
Other Gram-positive aerobic pathogens	3/4 (75) (19.4, 99.4)	3/4 (75) (19.4, 99.4)	2/3 (67) (9.4, 99.2)	8/11 (73) (39.0, 94.0)
All Gram-positive aerobic pathogens	35/40 (88) (73.2, 95.8)	26/29 (90) (72.6, 97.8)	10/13 (77) (46.2, 95.0)	71/82 (87) (79.2, 94.0)
Gram-negative aerobic pathogens	8/8 (100) (63.1, 100.0)	5/6 (83) (35.9, 99.6)	2/3 (67) (9.4, 99.2)	15/17 (88) (63.6, 98.5)
Anaerobic pathogens	0/0	0/0	1/1 (100) (2.5, 100.0)	1/1 (100) (2.5, 100.0)
All pathogens	39/44 (89) (75.4, 96.2)	28/32 (88) (71.0, 96.5)	12/15 (80) (51.9, 95.7)	79/91 (87) (79.9, 93.8)
No pathogens	13/14 (93) (66.1, 99.8)	4/7 (57) (18.4, 90.1)	9/10 (90) (55.5, 99.7)	26/31 (84) (66.3, 94.5)
**Final Follow-Up Visit**				
Staphylococcus aureus	32/37 (86) (71.2, 95.5)	21/28 (75) (59.0, 91.0)	8/11 (73) (39.0, 94.0)	61/76 (80) (71.3, 89.2)
MRSA	23/28 (82) (63.1, 93.9)	14/20 (70) (49.9, 90.1)	4/5 (80) (28.4, 99.5)	41/53 (77) (66.1, 88.6)
MSSA	10/10 (100) (69.2, 100.0)	7/8 (88) (47.3, 99.7)	4/6 (67) (22.3, 95.7)	21/24 (88) (67.6, 97.3)
Other Gram-positive aerobic pathogens	3/4 (75) (19.4, 99.4)	2/4 (50) (6.8, 93.2)	2/3 (67) (9.4, 99.2)	7/11 (64) (30.8, 89.1)
All Gram-positive aerobic pathogens	34/40 (85) (73.9, 96.1)	22/29 (76) (60.3, 91.4)	10/13 (77) (46.2, 95.0)	66/82 (80) (71.9, 89.1)
Gram-negative aerobic pathogens	8/8 (100) (63.1, 100.0)	5/6 (83) (35.9, 99.6)	2/3 (67) (9.4, 99.2)	15/17 (88) (63.6, 98.5)

Visit/Pathogen	Number of Successes/Number of Subjects (Clinical Success Rate [%]) (95% CI)			
	**750 mg q12h N=58**	**1000 mg q12h N=39**	**1000 mg q8h N=25**	**Total N=122**
Anaerobic pathogens	0/0	0/0	1/1 (100) (2.5, 100.0)	1/1 (100) (2.5, 100.0)
All pathogens	38/44 (86) (76.2, 96.5)	24/32 (75) (60.0, 90.0)	12/15 (80) (51.9, 95.7)	74/91 (81) (73.3, 89.3)
No pathogens	12/14 (86) (57.2, 98.2)	4/7 (57) (18.4, 90.1)	8/10 (80) (44.4, 97.5)	24/31 (77) (62.7, 92.1)

[a]CI, confidence interval; MRSA, methicillin-resistant *S. aureus*; MSSA, methicillin-susceptible *S. aureus*; q8h, every 8 hours; q12h, every 12 hours.

Note: A patient was counted only once in each pathogen category and may have been counted in multiple categories.

Supplement Figure 1. Component utility functions for (A) Effective cure rate, (B) Withdrawal rate, and (C) Contour plot of the utility function, which is the product of the two component utility functions [a,b]

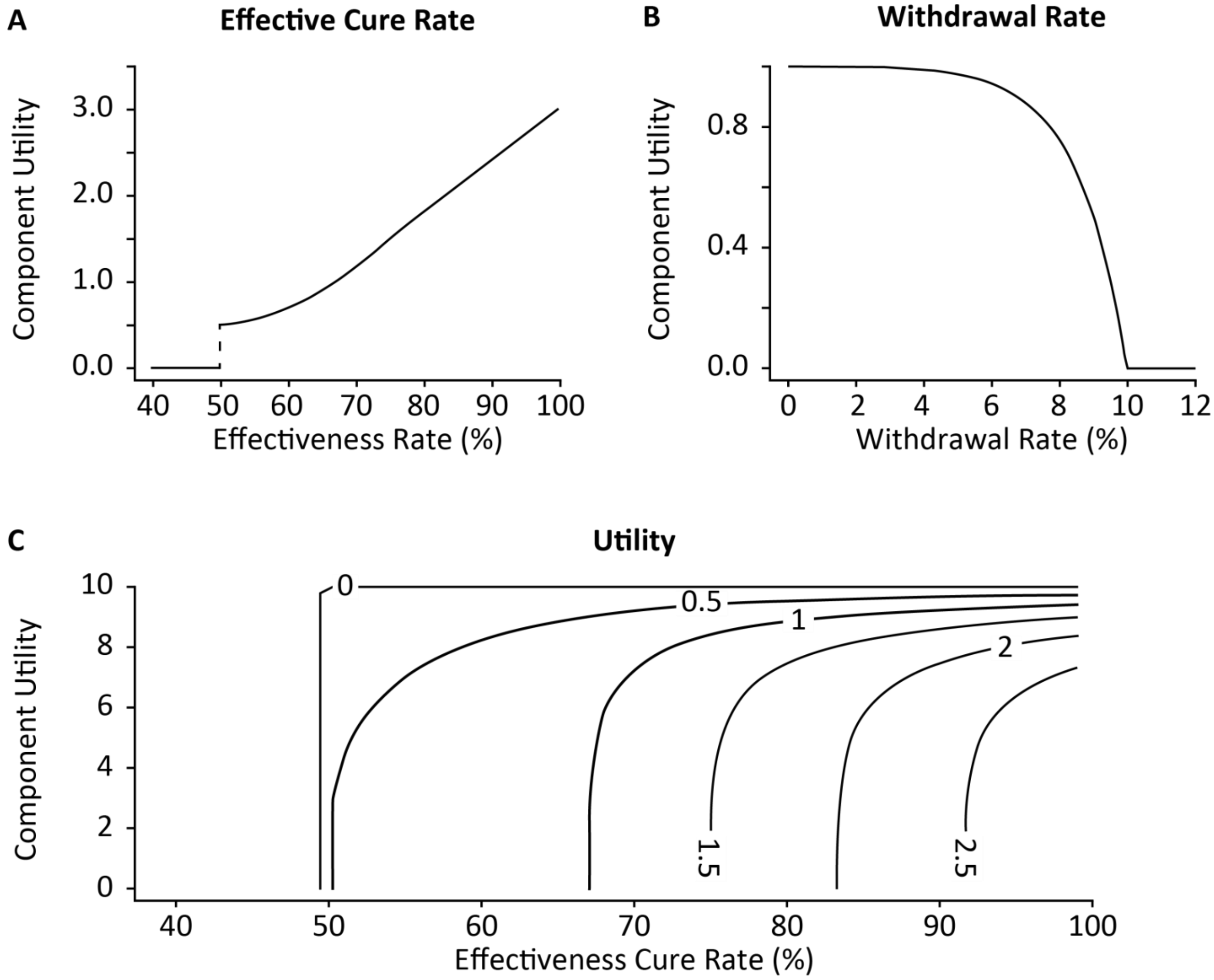

[a]The cure rate component of the utility function, which gives a score of 0 to cure rates below a clinically meaningful threshold of 50% and increasing utility for rates above the threshold.

[b]The withdrawal rate component of the utility function, gives “full score” to withdrawal rates <2.5%, then decreasing “scores” above 2.5%, to “no score” for withdrawal rates of ≥10%.
